# Sleep Patterns and Associated Insomnia in Junior and Senior Medical Students: A Questionnaire-Based Cross-Sectional Study

**DOI:** 10.1055/s-0043-1776731

**Published:** 2024-02-05

**Authors:** Ghady Dhafer Alshehri, Ahlam Ahmed Almahmoudi, Afnan Abdullah Alsaif, Bashayer Hassan Shalabi, Hana Zuhair Fatani, Fatima Hassan Aljassas, Dania Wazen Alsulami, Faris Alhejaili, Faisal Zawawi

**Affiliations:** 1 Department of Otolaryngology-Head & Neck Surgery, Faculty of Medicine, King Abdulaziz University, Jeddah, Saudi Arabia; 2 Department of Medicine, Division of Sleep Medicine, Faculty of Medicine, King Abdulaziz University, Jeddah, Saudi Arabia

**Keywords:** Athens Insomnia Scale, Pittsburgh Sleep Quality Index, medical students, sleep quality

## Abstract

**Introduction** Appropriate quality and quantity of sleep are critical for good mental health, optimal body functioning, memory consolidation, and other cognitive processes.

**Objectives** To evaluate the sleeping patterns of medical students in Saudi Arabia and their relationships with psychological distress.

**Methods** This was a cross-sectional, self-administered, questionnaire-based study. The study included medical students from a university in Jeddah, Saudi Arabia. The Pittsburgh Sleep Quality Index (PSQI) and the Athens Insomnia Scale (AIS) were used to evaluate the prevalence and burden of inadequate sleep quality and insomnia in the participants.

**Results** The majority of the participants was women (76.6%). Furthermore, most participants (96.2%) were aged between 18 and 24 years old, while 54.4% of the participants were in their senior year. According to the AIS scores (mean: 15.85 ± 4.52), 98.7% of the participants exhibited insomnia symptoms. The PSQI scores (mean: 9.53 ± 5.67) revealed that 70.5% of the participants had poor sleep quality. Students in their fundamental and junior years had significantly higher percentages of insomnia symptoms and poor sleep quality compared with students in their senior years.

**Conclusion** The prevalence of insomnia and poor sleep quality is high among medical students. Therefore, appropriate strategies for early detection and intervention are warranted.

## Introduction

Sufficient and high-quality sleep are important factors for good mental health and proper functioning of the human body as well as for memory consolidation and other cognitive activities. 1 The average sleep time of an adult is from 7 to 9 hours. However, this varies among individuals. Furthermore, owing to intensive academics and heavy clinical practice, medical students are at risk of establishing sleep impairment. 2

Medical students experience tremendous academic pressure and stress. 3 They do not prioritize sleep in the face of academic challenges and reduce their sleeping hours to modify and manage their workload and studies. Consequently, they have a higher chance of developing poor sleep patterns and disruptions, particularly prior to exams 4 ; this is linked to a higher risk of psychological morbidity and burnout. 5 Sleeplessness and extreme daytime sleepiness are the most frequent symptoms of poor sleep patterns among medical students. 6

A common sleep problem known as insomnia is characterized by difficulty falling or staying asleep despite having favorable opportunities and circumstances to do so. It is accompanied by symptoms such as irritability or weariness while awake. The underlying etiology of insomnia is multifaceted and involves genetic, environmental, behavioral, and physiological factors, all of which contribute to a hyperarousal state. 7 As a result, insomnia is common, affecting up to 30% of the general population. 8

Notably, several research studies have been done on the sleep patterns of medical students. One of these was a cross-sectional study conducted in Portland (United States of America) that assessed sleep quality and hygiene; it was discovered that the sleep quality of medical students was significantly improved poorer than that of healthy adults. 2 Furthermore, a cross-sectional study conducted in Brazil in 2016 analyzed and contrasted the subjective quality of sleep among medical students across different phases of their medical training. The comparison revealed that students in their 1 ^st^ and 2 ^nd^ years had poorer sleep quality and more daytime dysfunction compared with students in other years. 9 Similarly, other scientific evidence and current thought have confirmed the validity of the link between the first two years in medical school and poor sleep patterns, as students tend to have poorer sleep quality and more daytime dysfunction. 2 4 5 10

However, despite numerous studies on the sleep quality of medical students, no study has compared the sleep quality among junior and senior medical students. The present study aimed to compare the sleep patterns and incidence of insomnia between junior and senior medical students in Saudi Arabia. We also aimed to determine the relationship between these patterns and psychological distress.

## Methods

### Participants

The participants were medical students who were actively attending medical school at the largest university in Saudi Arabia. In this school, students receive 6 years of medical education and complete 1 year of internship thereafter. Students are admitted to the faculty of medicine directly after completing their high school education; therefore, the mean admission age is between 17 and 18 years old. The participants were divided into two groups: junior medical students (those in the 1 ^st^ , 2 ^nd^ , and 3 ^rd^ years of medical school) and senior medical students (those in their 4 ^th^ , 5 ^th^ , and 6 ^th^ years of medical school).

### Study Design, Setting, and Timeframe

This was a cross-sectional study that was performed in Jeddah, Saudi Arabia, from January 2022 to March 2022.

### Data Collection Instruments

A standardized and anonymized three-part questionnaire was distributed as a Google form among the participants to assess their sleep patterns and experience with insomnia. The demographic data of the participants' were collected in the first section, while the second part comprised the Pittsburgh Sleep Quality Index (PSQI), and the third part included the Athens Insomnia Scale (AIS).

The PSQI is a self-administered survey that enables a comprehensive evaluation of the sleep quality of the responder in the past month. 11 It has been proven effective in both clinical and nonclinical communities. 12 13 It consists of 19 questions divided into 7 sections that evaluate the following sleep parameters: quality, latency, total time of sleep, routine sleep effectiveness, sleep disruption, need for sleeping medicines, and daytime somnolence. Scores for each category range from 0 to 3 (0, not during the previous month; 1, fewer than once weekly; 2, once or twice a week; and 3, three or more times a week). The scores for these 7 elements are added together to produce a total score that ranges from 0 to 21. Poorer sleep quality is reflected by higher scores. The distinction between poor sleepers (> 5) and excellent sleepers (≤5) was made using a global cutoff score of 5. 11 14

The AIS is a self-administrated psychometric tool with eight elements 15 divided into two groups. The first five items deal with the nighttime symptoms of the respondent (difficulty falling asleep, remaining asleep, and getting up too early), while the final three deal with the effects of any reported sleep disturbances during the day. This effect considers how the respondent subjectively perceives their level of well-being, functional abilities, and daytime somnolence. An ordinal scale of 0 to 3 is used to rate each item in the AIS, with 0 and 3 denoting “no issue at all” and “extremely major concern,” respectively. If the respondent reported having problems falling asleep a minimum of three times a week during the preceding month, they had to evaluate each item positively. A cutoff score of ≥ 6 was used as the lowest score required to demonstrate the presence of insomnia symptoms, with the highest total score of 24 reflecting the most severe manifestations of insomnia.

### Research Ethics

The Ethical Committee of our institution approved the present investigation, and informed consent was obtained from the participants included in the survey.

### Data Analysis

Data were statistically analyzed using IBM SPSS Statistics for Windows, Version 26.0 (IBM Corp., Armonk, NY, USA). Using the chi-squared test, qualitative data, presented as numbers and percentages, were evaluated to determine the correlations between the variables. Numerical data are presented as means and standard deviations (SDs). Furthermore, nonparametric data were compared using Spearman correlation analysis. A *p* -value < 0.05 was considered statistically significant.

## Results

Out of the 397 participants, 76.6% were women, and 96.2% were Saudi nationals. The vast majority of participants (96.2%) was between 18 and 24 years old. Among them, 54.4% were in their senior years, and 26.7%, in particular, were in their 6 ^th^ academic year. The mean AIS and PSQI scores were 15.85 ± 4.52 and 9.53 ± 5.67, respectively ( Table 1 ).

**Table 1:** Distribution of the participants according to their characteristics and mean scores on the AIS and PSQI

Variable	*n* (%)
**Age (years old)**	
< 18	4 (1)
18–24	382 (96.2)
25–30	11 (2.8)
**Sex**	
Female	304 (76.6)
Male	93 (23.4)
**Nationality**	
Saudi	382 (96.2)
Non-Saudi	15 (3.8)
**Academic year**	
Fundamental year	88 (22.2)
Second	56 (14.1)
Third	37 (9.3)
Fourth	44 (11.1)
Fifth	66 (16.6)
Sixth	106 (26.7)
**Academic year category**	
Junior	181 (45.6)
Senior	216 (54.4)
**Marital status**	
Single	390 (98.2)
Married	5 (1.3)
Divorced	2 (0.5)
Mean AIS score	15.85 ± 4.52
Mean PSQI score	9.53 ± 5.67

Abbreviations: AIS, Athens Insomnia Scale; PSQI, Pittsburgh Sleep Quality Index.

**Fig. 1:**
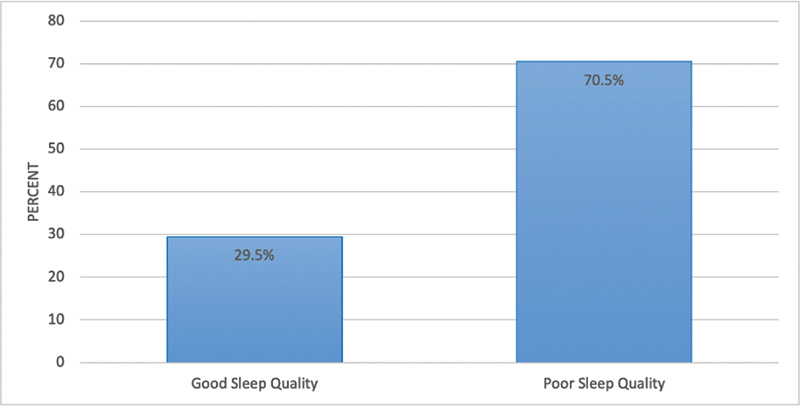
Percentage distribution of the participants according to sleep quality (determined using the Pittsburgh Sleep Quality Index scores).

**Fig. 2:**
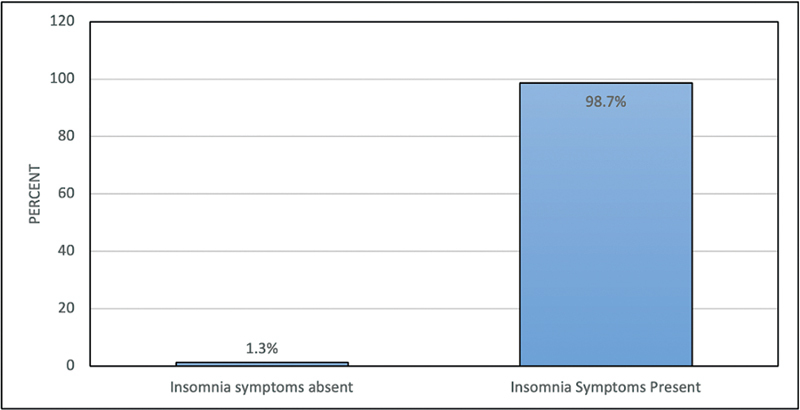
Percentage distribution of the participants according to insomnia symptoms (based on the Athens Insomnia Scale scores).

Based on the PSQI scores, 70.5% of individuals reported having poor sleep quality ( Fig. 1 ). Moreover, based on the AIS scores, most participants (98.7%) had symptoms of insomnia ( Fig. 2 ).

There was no significant relationship between the sleep quality of the participants and their age, sex, nationality, and marital status ( *p* ≥ 0.05; Table 2 ).

**Table 2:** Relationship between the participants' sleep quality (based on the PSQI) and their characteristics

Variable	Sleep quality		χ ^2^	*p-value*
	Good	Poor		
	*n* (%)	*n* (%)		
**Age (years old)**				
< 18	0 (0.0)	4 (100)		
18–24	113 (29.6)	269 (704)	1.92	0.382
25–30	4 (36.4)	7 (63.6)		
**Sex**				
Female	94 (30.9)	210 (69.1)	1.31	0.252
Male	23 (24.7)	70 (75.3)		
**Nationality**				
Saudi	115 (30.1)	267 (69.9)	1.95	0.162
Non-Saudi	2 (13.3)	13 (86.7)		
**Marital status**				
Single	114 (29.2)	276 (70.8)	0.68	0.711
Married	2 (40)	3 (60)		
Divorced	1 (50)	1 (50)		

Abbreviations: PSQI, Pittsburgh Sleep Quality Index; χ ^2^ , chi-squared.

**Fig. 3:**
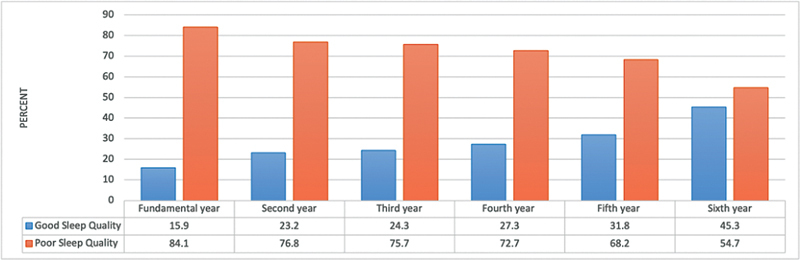
Relationship between sleep quality of the participants and their academic year N.B.: (χ ^2^ = 22.34; *p* < 0.001).

**Fig. 4:**
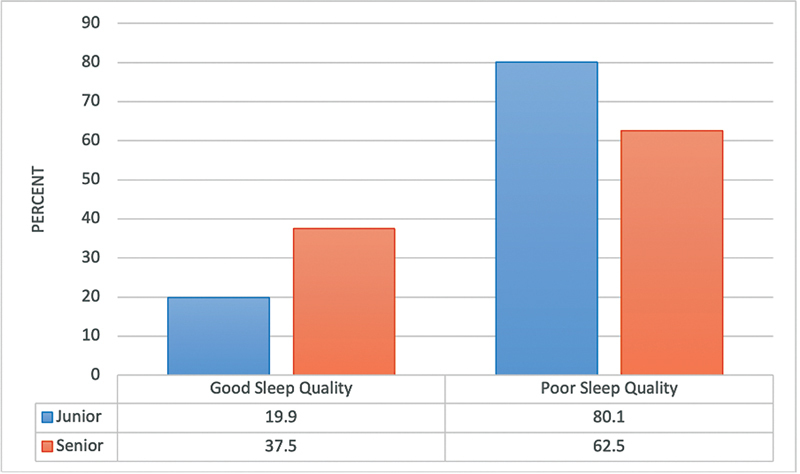
Relationship between sleep quality of the participants and their academic level N.B.: (χ ^2^ = 14.69; *p* <0.001).

A significantly higher percentage of students in the fundamental academic year ( Fig. 3 ) and of junior students ( Fig. 4 ) exhibited poor sleep quality ( *p* ≤ 0.05).

No association was discovered between the age, sex, nationality, or marital status of the participants and the presence of insomnia symptoms ( *p* ≥ 0.05; Table 3 ).

**Table 3:** Relationship between the participants' insomnia symptoms (based on the AIS score) and their characteristics.

Variable	Insomnia symptoms		χ ^2^	*p-value*
	Not present	Present		
	*n* (%)	*n* (%)		
**Age (years old)**				
< 18	0 (0.0)	4 (100)	0.19	0.905
18–24	5 (1.3)	377 (78.7)		
25–30	0 (0.0)	11 (100)		
**Sex**				
Female	4 (1.3)	300 (98.7)	0.03	0.856
Male	1 (1.1)	92 (98.9)		
**Nationality**				
Saudi	5 (1.3)	377 (98.7)	0.19	0.656
Non-Saudi	0 (0.0)	15 (100)		
**Marital status**				
Single	5 (1.3)	385 (98.7)	0.09	0.956
Married	0 (0.0)	5 (100)		
Divorced	(0.0)	2 (100)		

Abbreviations: AIS, Athens Insomnia Scale; χ ^2^ , chi-squared.

**Fig. 5:**
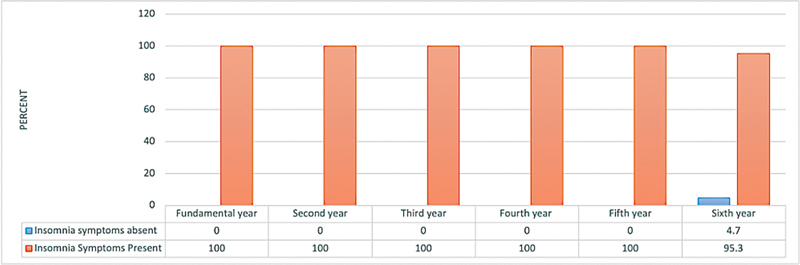
Relationship between the insomnia symptoms of the participants (based on the Athens Insomnia Scale score) and their academic year N.B.: (χ ^2^ = 13.9; *p* = 0.016).

**Fig. 6:**
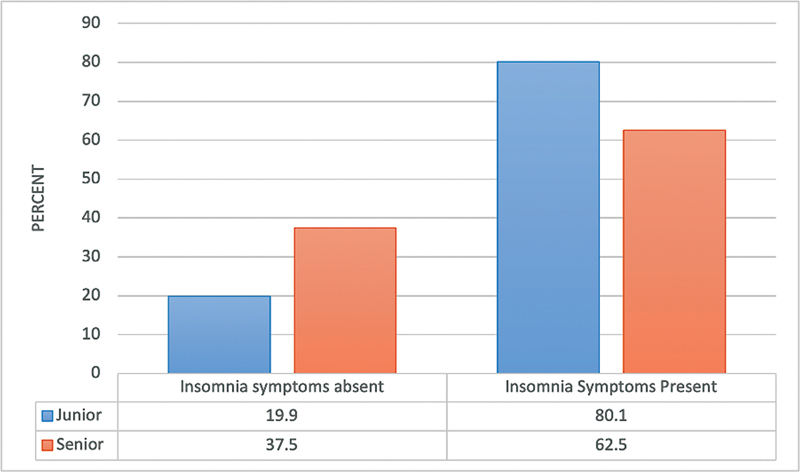
Relationship between insomnia symptoms of the participants (based on the Athens Insomnia Scale score) and their academic level N.B.: (χ ^2^ = 4.24; *p* = 0.039).

The prevalence of insomnia symptoms was significantly higher among students from the fundamental year to the 5 ^th^ year than among students in the 6 ^th^ year (p ≤ 0.05; Fig. 5 ). In addition, insomnia symptoms were significantly more common in junior students than in senior students ( *p* ≤ 0.05; Fig. 6 ). A highly significant positive correlation ( *p* ≤0.05) was found between the PSQI and AIS scores.

**Fig. 7:**
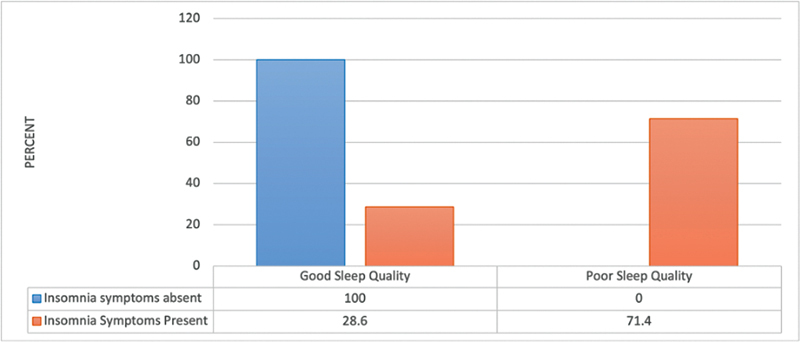
Relationship between the presence of insomnia symptoms and sleep quality N.B.: (χ ^2^ = 12.11; *p* < 0.001).

Furthermore, the number of students with poor sleep quality was higher in those who had insomnia symptoms than in those who did not ( *p* ≤0.05). ( Fig. 7 )

## Discussion

Insomnia and poor sleep quality have significant long-term effects on a person's health and general performance. These are prevalent in the Saudi population; thus, more awareness is required. 16 Numerous studies, in particular, have revealed that medical students frequently have poor sleep quality. 17 Medical students aim to reach the highest standards of excellence, often pushing themselves beyond their limits; hence, they may struggle with numerous stressors, which may affect their sleep hygiene and quality. Therefore, it is crucial to detect poor sleep patterns and insomniac tendencies that necessitate interventions in such groups. 18 19

We found that ∼ 70% of the sample size in the present study had poor sleep quality. This result is consistent with the findings of a study conducted in Saudi Arabia using the same questionnaire, which found that 76% of medical students had poor sleep quality as a prevalence. 4 Poor sleep quality could potentially contribute to the vulnerability of medical students to stress as the need to study extensive academic material in a short time leads to prolonged hours of studying and an early morning schedule, which results in insufficient sleep. The prevalence of poor sleep observed in the present study is consistent with that reported in two other studies conducted in Pakistan 18 and in Spain. 19 According to the findings of our study, insomnia was the main indicator of inadequate sleep in 98.7% of the participating medical students. Similarly, another research conducted in Saudi Arabia discovered that 60% of the students were incapable of falling asleep within half an hour of going to bed and experienced several nocturnal awakenings. 20

The current study found no statistically significant link between age and sleep quality. Conversely, a study by Giri et al. on students from a deemed university in India revealed that age and sleep quality have a significant positive association. 21 This difference may be due to the inclusion of postgraduate physicians in their study, who may experience more stress from being on call during the night. Furthermore, our findings showed that there was no statistically significant association between sex and sleep quality, which is in accordance with the results of a comparable study carried out at Al-Qassim, where there were no sex differences in the findings of the study on sleep quality ( *p* < 0.219). 22 Moreover, this study revealed an insignificant association between the quality of sleep and marital status, which is consistent with a study conducted in Iran that showed no significant association between sleep quality and marital status ( *p* < 0.638). 22

University students are at a high risk of developing sleep disorders, and medical students are particularly susceptible to them. 23 24 Furthermore, sleep quality may be affected by difficulties in psychological well-being, such as the presence of stress, anxiety, and depressive symptoms). 4 25 Our study found that junior medical students had poorer sleep quality than senior medical students; this supports the results of a previous study. 3 Collectively, these findings may indicate improved mechanisms of coping with their curriculum. 3 Moreover, in the 1 ^st^ year of medical college, there is a significant transformation that tests the coping strategies of the students towards stressors that accompany a wide range of obstacles. 26

The findings of our study indicated that there were no significant differences in the quality of sleep between men and women, and age and marital status did not significantly affect sleep quality. Conversely, a study performed at the Nishtar Medical University, Pakistan, concluded that married females and older adults had notably higher rates of insomnia. 27 This discrepancy may be due to the inclusion of high-school students and the variations in the curriculum, studying hours, and extracurricular activity between students in high school and medical students. Moreover, most high school students are usually unmarried. 27 Our study revealed that most medical students had insomnia symptoms, with 100% of students in the fundamental year through the 5 ^th^ year and 95.3% of students in the 6 ^th^ year exhibiting insomnia symptoms.

The heavy stress and pressure that medical students experience can directly impact their sleep. Compared with senior students, more junior students experienced insomnia symptoms (62.5 versus 80.1%). Therefore, fewer senior students experienced insomnia symptoms when compared with junior students. This may be explained by the tendency for junior students to have greater anxiety before beginning medical school because they are under greater pressure to cope with the new lifestyle modifications, maintain their GPA, and excel in their performance.

Nevertheless, these findings show a significant prevalence of insomnia symptoms among medical students. We also discovered a positive correlation between poor sleep quality and insomnia in our sample population. Another study reported that individuals with poor sleep quality were more prone to experience insomnia symptoms. 28

Despite using validated questionnaires and a sizable sample size, the present study has some limitations. Its cross-sectional design is one of its drawbacks. In addition, the present study was limited to the students at one university; thus, the sample may not represent the general student population. Furthermore, the sleep patterns were based on the “self-reports” of the students rather than on an objective assessment, which may have led to bias. Further research should be conducted at other colleges and should focus on the effects of confounding factors such as unhealthy weight, smoking status, energy-drink consumption, and sleep deprivation on psychological stress.

## Conclusion

Our study investigated insomnia and sleep patterns among medical students in Saudi Arabia. Our results revealed a high prevalence of poor sleep quality and insomnia in this population. A counseling center that conducts stress management programs in colleges, screening tools for early detection, and educational courses on achieving sufficient sleep within a stressful environment are required.
